# The Field of Science

**Published:** 1893-11-18

**Authors:** 


### The Field of Science.

During the recent epidemic at Greenwich Workhouse it was observed that none of the habitual smokers were attacked by the complaint which had laid low the other inmates of the institution. Theoretically we have long spoken of the bactericidal value of tobacco, but in this instance it is exemplified to us more practically in a definite form. This has given rise to a suggestion from some quarters, which, however, we do not endorse, that during serious epidemics smoking merits as much consideration as vaccination, or, indeed, as do the other recognized prophylactic means of combating the contagion.

For many years past now the Lariboisière Hospital in Paris has contributed its quota in the manufacture of cigarette papers for the Parisian capital. It appears that the cotton wool and lint, after being used in the wards of the institution, were regarded as the special perquisites of the servants, who sold them to manufacturers of the particular industry to which we have referred. This proceeding, which has now been put a stop to, was carried out *sub rosa*, and it may readily be surmised was never widely known, or perhaps consumers of Parisian cigarettes would have been fewer in number.

Messner's investigations recently carried on with a view to discover whether rifle bullets are capable of carrying infection, show beyond all doubt that this is the case. Bullets which are purposely brought into contact with micro-organisms and are then discharged in the usual manner, carry the microbes with them into whatsoever material they find a lodgment. Materials which are thus previously infected, and subsequently fired through by a bullet, act in the same injurious manner, but the microbe itself loses no vitality in the process. This interesting experiment is used as an explanation why gunshot wounds prove so often fatal—the shot, it is said, in first striking the earth, may carry infection from the tetanus organism, and then entering the flesh, be the means of producing lockjaw and other fatal ills.

Dr. Wetterstand, of Stockholm, not only adds further confirmatory testimony in this direction, but particularizes the advantages derived from long sleep. In the case of epileptics, nine or ten persons, he reports, who have come under his own personal treatment, have had no recurrence of fits since, now many years ago, he employed the remedy, which he advocates. He also reports successful treatment in some instances for dipsomaniacs. Turning to our own country, Dr. Bramwell has much to say as to its power. But at the same time it should be understood that he does not regard hypnotism as a rival in any sense to ordinary medicine, or as a specific for all diseases, but simply as an additional weapon for the combat of disease, with which he considers every medical man should be acquainted, and which, if they so wished, they should be permitted to employ.

Perhaps the principal feature which characterised the Norwegian Medical Congress of this year was the locale of its meeting. The sessions were arranged to be held on a large steamboat expressly chartered for the meetings. Two salient points in this arrangement recommend it to our attention. In the first place, modern hygiene through this means was not only preached but put into practice, for throughout the Congress there was a complete avoidance of the pernicious atmosphere of crowded rooms. In the second place, the assembly being held in movable quarters, possessed the further advantage of being able to proceed from place to place along the coast, and thus enlarge the area of its usefulness.

Hypnotic treatment for disease is now a recognised agency in Russia, and the stringent prohibitions against the therapeutic employ of the "influence" are now abolished, and, with certain restricting regulations, the medium has received State sanction. It is still a matter of dispute elsewhere whether the case in its defence should be allowed a hearing. There is a practical demonstration in many countries, as in the case of Russia above quoted, wherein the advantages derived from hypnotic suggestion are confidently asserted. International statistics on this subject are interesting reading, since they are written in good faith and published under the ægis of a number of medical experts. Amongst a mass of statistics we are told that, out of 8,705 cases noted by 15 observers in nine different countries, 6 per cent. only of persons are quoted as failing to come under the influence of hypnotism.

Business men (and business women, too) show but little care for their eyesight when they wantonly give themselves up to the pernicious habit of reading their fifth editions on their return journey home after a long day's work, by the varied means of conveyance offered them in train, cab, or omnibus. This latter vehicle in particular it is which lends itself least kindly to the habit against which we give a gentle warning. The shifting, uncertain light of an omnibus is wholly insufficient for any continuous reading, unless, indeed, a very severe strain be put on the organs of vision themselves. For how is it possible to focus so as to keep pace with the momentary changes which pass over the page from without the windows. The flash light of a chemist's shop, the thousand candle power of the electric current, the hundred illuminations which go to make up the lighting of the City streets, intermixed, as these are, by long intervals in which the semi-obscurity of the metropolitan stage coach dominates alone—all these fluctuations playing on small print are trying to eyes at the best of times; and at the end of the day the tax on nerve and mind is greater; neither, indeed, is strong enough to combat the ill-effects which inevitably accrue to those who habitually digest their evening news under these circumstances.

